# Fine-needle Aspiration Cytology to Identify a Rare Mimicker of Breast Cancer: Plasma Cell Mastitis

**DOI:** 10.1055/s-0038-1666809

**Published:** 2018-07-09

**Authors:** Carlos Manuel Ortiz-Mendoza, Norma Alicia Acosta Sánchez, Arturo Catarino Dircio

**Affiliations:** 1 Department of Surgery, Instituto de Seguridad y Servicios Sociales de los Trabajadores del Estado (ISSSTE) Hospital General Tacuba, Mexico City, Mexico; 2 Department of Pathology, ISSSTE Hospital General Tacuba, Mexico City, Mexico

**Keywords:** cancer, breast, breast cancer, fine needle aspiration biopsy, granuloma, mammography, mastitis, câncer, mama, câncer de mama, biopsia por aspiração com agulha fina, granuloma, mamografia, mastite

## Abstract

There are rare benign diseases that can mimic malignant breast neoplasms in the clinical exam and in mammography. We evaluated the contribution of an accessible procedure to most clinicians, the fine-needle aspiration cytology, to identify a rare mimicker of malignant breast neoplasms. A type 2 diabetic 85-year-old female presented with a 6-month history of a left breast lump. The physical exam and mammography were compatible with breast cancer. Nevertheless, after fine-needle aspiration cytology, the diagnosis was plasma cell mastitis. Once this rare diagnosis was established, the tumor was extirpated, and the final histologic diagnosis corroborated chronic plasma cell mastitis. The patient's postoperative evolution was uneventful, and no other treatment was needed. Fine-needle aspiration cytology could be a valuable tool to identify rare mimickers of malignant breast neoplasms.

## Resumo

Existem doenças benignas raras que podem mimetizar neoplasias malignas de mama no exame clínico e na mamografia. Avaliamos o valor de um procedimento acessível para a maioria dos clínicos, a citologia por aspiração com agulha fina, para identificar um imitador raro de neoplasias malignas de mama. Uma mulher de 85 anos com diabetes tipo 2 apresentou histórico de 6 meses de um nódulo no seio esquerdo. O exame físico e a mamografia foram compatíveis com câncer de mama. No entanto, após realizar uma citologia por aspiração com agulha fina, o diagnóstico foi mastite celular plasmática. Uma vez que este diagnóstico raro foi estabelecido, o tumor foi extraído e o diagnóstico histológico final corroborou a mastite crônica das células plasmáticas. A paciente teve uma boa evolução pós-operatória, e nenhum outro tratamento foi necessário. A citologia por aspiração com agulha fina pode ser uma ferramenta valiosa para identificar os raros mimetizadores de neoplasias malignas da mama.

## Introduction

Breast cancer is a major health problem worldwide and its number is growing.^1^ Rarely, there are benign diseases than can mimic malignant invasive breast tumors in clinical exam and mammography.^2^ Thus, different forms of biopsy are needed to confirm this diagnosis; this is called the triple test score.

Fine-needle aspiration cytology (FNAC) is an office procedure, involving low-cost and usual medical supplies; this method is accessible to most clinicians. With appropriate training and experience of clinicians and pathologist, its rate of diagnostic accuracy is high: sensitivity (92.7%) and specificity (94.8%).^3^ The value of FNAC in recognizing a rare mimicker of breast cancer in clinical and mammographic backgrounds is herein assessed.

## Case Description

**Fig. 1:**
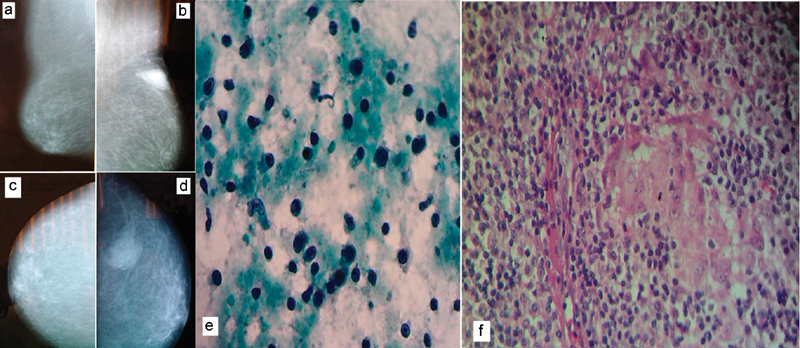
(**a**) Mammography: Mediolateral oblique view of the right breast. (**b**) Mediolateral oblique view of the left breast. (**c**) Craniocaudal view of the right breast. (**d**) Craniocaudal view of the left breast. (**e**) Fine-needle aspiration cytology specimen of the tumor (Papanicolaou stain) showing many plasma cells. (**f**) Microphotography of the surgical specimen (hematoxylin and eosin [H and E] stain, 100x) showing several plasma cells and giant cells.

An 85-year-old woman with type 2 diabetes presented with a 6-month history of a left breast lump. Practicing of self-breast exam and mammography was denied by the patient. At physical exam, the right breast and both axillae were normal; however, the left breast had a hard and painless 3 cm lump in the external upper quadrant. A mammography showed a left breast tumor with irregular margins, distorting the regional breast architecture; the result of the mammography led to the diagnosis of a category V lesion, according to the breast imaging reporting and data system (BI-RADS) (Fig. 1b and d). With these findings, a left breast cancer (T2 N0) was suspected. To confirm the clinical and radiological impression, a FNAC was performed; unexpectedly, the cytological diagnosis was plasma cell mastitis (Fig. 1e). To support this extremely rare cytological diagnosis, a wide tumor resection was arranged. The final histopathological diagnosis of the surgical specimen was chronic plasma cell mastitis (Fig. 1f). The patient had an uneventful postoperative course and did not need any other treatment.

## Discussion

In this case, the FNAC was an appropriate tool to identify a rare mimicker of breast cancer: the plasma cell mastitis. Fortunately, the FNAC is an accessible diagnostic method to most clinicians. After performing a search in different repositories (PubMed, Lilacs, Scopus, and Google scholar), we found that the potential usefulness of FNAC to identify granulomatous mastitis, as shown in our case, was in accordance with the study of Akcan et al.^4^ However, two different groups found opposite results about the FNAC's utility, and they emphasize the great difficulty to differentiate a carcinoma from a granulomatous mastitis with this kind of biopsy.^5^ ^6^ A combination of the lack of adequate training and experience in FNAC, technique execution and interpretation is probably the answer to these contradictory findings.^3^

The presented patient was an 85-year-old female. There is inconsistent information concerning the age-group most affected by granulomatous plasma cell mastitis. We agree with Bhaskaran et al^7^ about plasma cell mastitis occurring in older women; nonetheless, there are two reports indicating that this kind of mastitis occurs more frequently in young females.^6^ ^8^ To increase the complexity of this issue, plasma cell mastitis is a rare form of mastitis, and its pathogenesis is not yet fully understood.^7^ ^8^ ^9^

Plasma cell mastitis belongs to a rare group of granulomatous breast diseases.^8^ Common opinion between experts of this field indicates that all forms of granulomatous mastitis can mimic breast cancer in clinical and radiological backgrounds.^2^ ^4^ ^5^ ^6^ ^7^ ^8^ This is why the triple test score is essential for clinicians when evaluating palpable breast tumors: physical exam, radiologic evaluation (mammography and/or echography), and a biopsy.^3^ As we can see in this case, the result of the biopsy changed the diagnosis dramatically. A careful history and a diligent physical exam are the first steps in identifying any disease; however, as with at all medical diagnostic tools, they have their own limitations and exactness.

## Conclusion

Fine-needle aspiration cytology is a valuable diagnostic tool. It can detect rare mimickers of malignant breast tumors classified as BI-RADS category V, thus, radically changing the course of treatment.
